# Serum Cystatin C Predicts Mortality in HBV-Related Decompensated Cirrhosis

**DOI:** 10.1155/2019/7272045

**Published:** 2019-03-04

**Authors:** JianPing Wu, QianXia Wu, MinYi Wu, WeiLin Mao

**Affiliations:** Department of Clinical Laboratory, The First Affiliated Hospital, College of Medicine, Zhejiang University, Hangzhou, Zhejiang 310003, China

## Abstract

**Background:**

Some studies have reported that renal dysfunction is associated with poor prognosis in cirrhotic patients. Serum cystatin C (CysC) is an accurate biomarker for early renal dysfunction. This study aimed to assess the prognostic value of serum CysC levels in patients with hepatitis B virus-related decompensated cirrhosis (HBV-DeCi).

**Methods:**

This retrospective study included 75 subjects who had been diagnosed with HBV-DeCi. The association between serum CysC and prognosis was estimated by receiver operating characteristic curve analysis and a multivariable logistic regression model.

**Results:**

Serum CysC levels were higher in nonsurvivors than in survivors and were positively correlated with model for end-stage liver disease (MELD) scores. In multivariate analysis, CysC and the MELD score were independent prognostic factors in all HBV-DeCi patients. However, only serum CysC was an independent factor predicting mortality in patients with normal creatinine levels.

**Conclusions:**

These data suggest that high serum CysC levels can be considered an independent biomarker of 3-month mortality in patients with HBV-DeCi.

## 1. Introduction

Liver fibrosis and cirrhosis are the main causes of morbidity and mortality in patients with hepatitis B virus (HBV) infection [1]. In China, the annual rate of progression from compensated cirrhosis to decompensated cirrhosis (DeCi) is between 1.5 and 5% [2]. Decompensated cirrhosis is defined by the appearance of clinical ascites, variceal bleeding, or hepatic encephalopathy (HE), and the prognosis is markedly worse; the 5-year survival rate is only 19-35% after decompensation [3].

In patients with advanced liver cirrhosis, portal hypertension usually causes an insufficiency of effective circulating volume and alters haemodynamics, leading to redistribution of blood flow in the kidney, water-sodium retention, a reduced glomerular filtration rate (GFR), and simultaneous compensatory activation of the endogenous sympathetic and renal vasoconstrictor systems. Thus, renal dysfunction is often an accompanying condition in patients with DeCi [4]. Previous data have shown that renal dysfunction is associated with poor prognosis in patients with liver cirrhosis [5, 6] and pretransplant renal dysfunction is also closely related to a worse survival rate after liver transplantation [7–9]. Many experts have found that renal function is superior to liver function in predicting the prognosis of DeCi patients [10–12]. Therefore, accurate and timely estimation of renal function is very important in patients with DeCi and can provide clinical evidence for early intervention treatment, which may help improve their clinical outcomes.

Cystatin C (CysC) is a cysteine proteinase inhibitor produced at a constant rate and freely filtered by the glomerular membrane. Several studies suggest that CysC is an effective indicator of mild renal dysfunction in contrast to classic biomarkers such as serum creatinine (Cr) or Cr-based formulae [5, 6]. Previous studies have indicated that CysC could be a useful biomarker for prognosis in patients with liver disease. For example, Yeon et al. found that CysC is a useful prognostic biomarker of mortality among patients with cirrhotic ascites [13]. Recently, Wan et al. reported that serum CysC plus total bilirubin can predict the 3-month mortality of HBV-ACLF patients [14]. In a recent study conducted in 2017, Markwardt et al. demonstrated that elevated baseline CysC is associated with poor outcomes among patients with acutely DeCi [15]. Despite these correlations, few studies have evaluated the association between serum CysC and outcomes in HBV-DeCi patients. We conducted this study to further elucidate the prognostic value of CysC regarding the 3-month mortality of patients with HBV-DeCi.

## 2. Materials and Methods

### 2.1. Study Population

**Figure 1:**
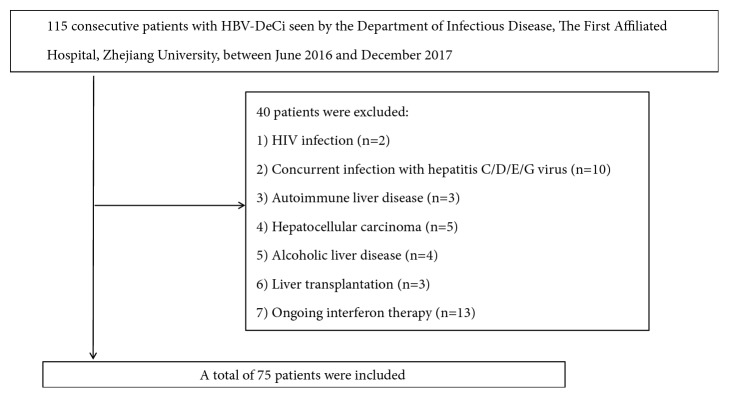
Flow chart of the enrolled participants.

We continuously analysed 115 patients with HBV-DeCi between June 2016 and December 2017. Patients had to be HBsAg positive, previously diagnosed with HBV-related compensated cirrhosis and now presenting clinical manifestations of decompensated liver disease for the first time. Liver decompensation was defined as the occurrence of complications, such as ascites, variceal bleeding, or HE [16]. The exclusion criteria included alcoholic liver disease, hepatocellular carcinoma or other malignancies, autoimmune disease, primary kidney disease, cardiovascular dysfunction, or coinfection with hepatitis C/D/E/G virus or human immunodeficiency virus; only patients without antiviral, interferon, or immunotherapy treatment 6 months prior to sampling were included in the study. All participants received antiviral therapy from the start date. Following the criteria above, 75 patients were enrolled. For each patient, retrospectively collected data, including patient demographics and clinical and laboratory variables, were abstracted from the medical records at baseline. The laboratory parameters included measurement of total protein, albumin, alanine aminotransferase (ALT), aspartate aminotransferase (AST), total bilirubin, serum Cr, serum CysC, and international normalized ratio (INR). All biochemical indices were measured using a Hitachi 7600 clinical analyser (Hitachi, Tokyo, Japan).The INR was analysed using the coagulation method with a Sysmex CS-2000i Analyser (Sysmex, Kobe, Japan). The normal adult reference range for serum CysC was 0.40–1.10 mg/L and normal serum Cr level was < 97 *μ*mol/L in the Hospital laboratory, respectively. The severity of liver dysfunction was estimated by Child-Pugh and Model for End-Stage Liver Disease (MELD) scores at the time of admission. In addition, serological indices (HBsAg, HBeAg, and anti-HBc) were retrospectively collected at baseline.  Figure 1 shows the 40 excluded patients and 75 patients who were ultimately included in this study. All patients were followed up for at least 3 months to identify the status of clinical outcomes.

The study was performed according to the Declaration of Helsinki; the procedures were approved by the Ethics Committee of the First Affiliated Hospital, School of Medicine, Zhejiang University.

### 2.2. Calculation of Scores

The Child-Pugh score was calculated according to the total bilirubin, albumin, INR, ascites status, and degree of HE [17].

The MELD score was calculated according to the following formula: MELD score = 3.78 × ln (total bilirubin, mg/dl) + 11.2 × ln (INR) + 9.57 × ln (Cr, mg/dl) + 6.43 [18].

### 2.3. Statistical Analysis

All continuous variables are presented as the means and standard deviation or median (25^th^-75^th^ percentiles). Categorical values are shown as percentages. The differences between nonsurviving patients versus surviving patients with HBV-DeCi were assessed with an independent sample* t*-test, the Mann-Whitney* U* test, or the chi-square test, as appropriate. Correlations between variables were examined using Spearman's correlation analysis. The prediction of in-hospital 3-month mortality by different variables was evaluated using area under the receiver operating characteristic (AUROC) curves. Independent predictors for mortality were identified using univariate and multivariate logistic regression analysis. Statistical analyses were performed using SPSS version 16 (SPSS Inc., Chicago, IL) and MedCalc version 15.2.1 software (MedCalc, Ostend, Belgium).* P* < 0.05 was considered to indicate significance.

## 3. Results

### 3.1. Demographic and Clinical Features

A total of 75 patients with HBV-DeCi were included. Demographic and clinical characteristics of the participants are presented in Table 1. Overall, 59 patients (78.7%) were male, and 16 (21.3%) were female; the mean age was 53.0 ± 11.0 years (range, 27–74 years). The presenting features of decompensation were ascites (n = 48, 64.0%), variceal bleeding (n = 22, 29.3%), hepatorenal syndrome (HRS) (n = 5, 6.7%), and HE (n = 2, 2.6%). The CysC level was positively correlated with the MELD score (*r *= 0.490,* P *< 0.01) and serum Cr level (*r *= 0.437,* P *< 0.01), but there was no correlation with the Child-Pugh score (*P* = 0.521). Moreover, we found that the level of CysC was not different between male and female patients (data not shown).

### 3.2. The Serum CysC Was Higher in Nonsurvivors Than in Survivors

During the follow-up, 25 patients died within 3 months. We compared the differences in clinical characteristics and variables between nonsurviving and surviving patients (Table 2). No significant differences were observed in age, gender, total protein, albumin, total bilirubin, ALT, or AST between the surviving and nonsurviving groups. Nonsurviving patients had a much higher CysC level than the surviving patients (median 1.52, interquartile ranges 1.12–2.12 versus 0.98, 0.87–1.18,* P*<0.01).

Furthermore, the nonsurvivors had a higher MELD score, serum Cr level, and INR than the surviving patients.

### 3.3. High Serum CysC Levels Indicate Poor Short-Term Outcomes in HBV- DeCi Patients

**Figure 2:**
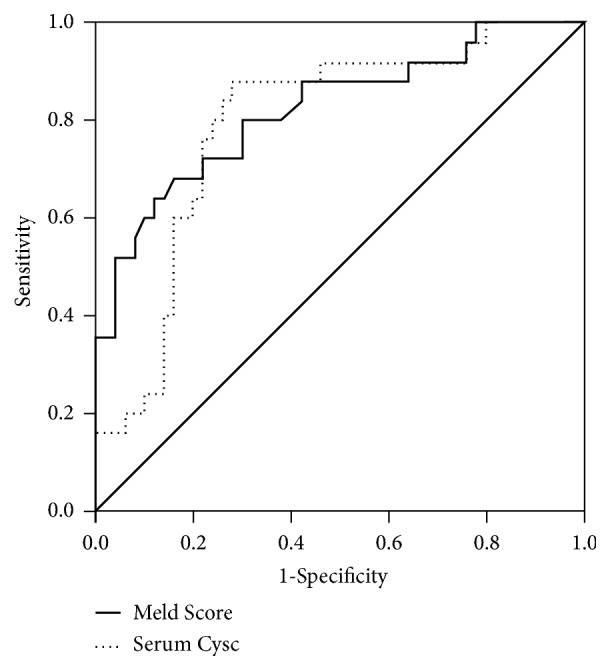
Receiver operating characteristic curve of serum CysC (▪▪▪▪◾) and MELD score (**—**) at admission for predicting 3-month mortality in HBV-DeCi patients.

During the follow-up period, 25 patients died (33.3%) from the following causes: upper gastrointestinal bleeding (n=8), HE (n=2), hepatic failure (n=4), HRS (n=10), and unknown (n=1). Univariate and multiple logistic regression analysis identified both MELD score and CysC as associated with mortality 3-month mortality in DeCi patients (Table 3). ROC curve analysis was performed to evaluate the relative efficiencies of the CysC and MELD scores for predicting mortality (Figure 2). The optimal cut-off values, sensitivity, and specificity were 17.8, 80.0%, and 76.0% for MELD and 1.35, 68.0%, and 84.0% for CysC, respectively. The AUC values in predicting mortality were 0.793±0.055 for the MELD score (95% CI: 0.684–0.878,* P*<0.01) and 0.823±0.054 for the CysC (95% CI: 0.718–0.902,* P*<0.01). CysC and MELD score predicted mortality with similar power (Z=0.429,* P*=0.668). In the current study, 55 patients had normal Cr levels (< 97 *μ*mol/L), including 43 males and 12 females aging from 27 years to 73 years; we further explored the predictors of these patients. The multivariate logistic regression analysis indicated that only CysC was an independent factor predicting mortality (Table 4).

## 4. Discussion

The current research was performed to evaluate the prognostic value of serum CysC, an accurate biomarker of renal function, in HBV-decompensated cirrhosis. Our results show that higher serum CysC levels were associated with increased mortality in patients with HBV-DeCi. Further multivariate analysis identified that serum CysC may serve as an independent predictive indicator of mortality.

The development of renal dysfunction in cirrhotic patients is related to adverse outcomes. Serum Cr has been used as an important indicator reflecting renal function over the past 50 years. Nevertheless, several reports have demonstrated that serum Cr is not a perfect indicator of renal function because it is altered by various nonrenal factors, such as body weight, race, gender, age, and muscle metabolism. Moreover, serum Cr has limited potential in the diagnosis of early renal dysfunction because it may not increase until renal function is already severely impaired [10, 19]. These factors are likely to make serum Cr levels misleading in predicting renal function. The present study demonstrated that serum CysC has a positive correlation with serum Cr (*r *= 0.437,* P *< 0.001). This result differs from those of Wan et al. [20], which indicated that serum CysC has no association with Cr levels. The reason for this discrepancy may be because their study population was composed of 56 patients with HBV-related ACLF, while we focused on HBV-DeCi patients and the differences in the stages of liver diseases of patients recruited may be associated with different outcomes. In recent years, some research has demonstrated that CysC is a valid alternative to Cr because it is free from extrarenal factors. In our study, serum CysC concentration was above normal value (≥1.10 mg/L) in 34 (45.3%) patients, suggesting that renal dysfunction may occur at the time of inclusion. In addition, serum CysC is positively related to the MELD score, and nonsurvivors had higher serum CysC than survivors. The MELD score was first published in 2000 to predict the survival of patients undergoing transjugular intrahepatic portosystemic shunts. Moreover, it has been used to assign the priority of liver transplantation candidates [18]. Our previous study reported that the MELD score was also associated with the prognosis of patients with HBV-related ACLF [21]. At present, our results showed that serum CysC levels were independently associated with mortality in all HBV-DeCi patients. In fact, the predictive power of the MELD score was slightly lower than that of the CysC. A possible explanation for this was that renal dysfunction is a well-known complication of advanced cirrhosis and renal functions are better predictors of survival than those routinely used to estimate hepatic function in these patients [10–12]. It is reported that Cr frequently overestimates kidney function in the cirrhotic population, particularly more often in patients with mild-to-moderate impairment. So, CysC maybe had better AUC compared to Cr-based MELD score. The Child-Pugh and MELD scores are the most commonly used scoring systems to evaluate the prognosis of HBV-related cirrhosis. The MELD score incorporates 3 laboratory variables, total bilirubin, INR, and serum Cr, and it is used to assess not only liver dysfunction but also renal dysfunction. The Child-Pugh score was calculated according to the total bilirubin, albumin, INR, ascites status, and degree of HE, and it primarily assesses dysfunction of the liver but not other organs. In a study comparing the ability of the two scoring systems to predict inpatient mortality in cirrhosis, the authors found that the MELD score was superior to the Child-Pugh score in predicting mortality at 3 months to 3 years [22]. In our study, the Child-Pugh score was not a risk factor for 3-month mortality, and this result was identical to that in a study by Fontana et al., who showed that the Child-Pugh score did not predict 6-month mortality in patients with HBV-DeCi [23]. Consistent with the data from Seo's group [5], our findings also indicated that only the serum CysC was an independent factor predicting the mortality rate in patients with normal Cr. It may be that most of the patients had normal Cr levels in our study, which may make the Cr-based MELD scoring system less powerful in predicting prognosis for these patients. One possible explanation is that the Cr level rises after serious renal damage. Our results suggest that mild-to-moderate renal dysfunction may occur in HBV-DeCi patients whose Cr levels are within the normal range. Hence, early and accurate assessment of renal function is very important; it may prevent or slow down progression and will help improve clinical management to reduce high mortality.

Our study has some limitations. First, the retrospective study design may have led to patient selection bias. Moreover, this was a single-centre study in China, and the sample size was not sufficient. In addition, it should also be noted that other Cr-based formulae, such as estimation of the GFR, were not examined in our patients. Therefore, we cannot confirm whether the CysC level is truly representative of renal function status. Thus, further studies with prospective and mechanistic designs need to be performed in multiple centres to verify the predictive value of CysC in patients with HBV-DeCi.

In summary, our findings demonstrate that serum CysC is objectively determined and would be a simple and inexpensive prognostic indicator in patients with HBV-DeCi. In the future, a prospective clinical trial will be necessary to validate the current findings.

## Figures and Tables

**Table 1:** Baseline clinical and laboratory characteristics of the study population.

	HBV-DeCi patients (n=75)
Gender (male/female)	59/16
Age (y)	53.0±11.0
Total protein (g/L)	58.8±8.1
Albumin (g/L)	28.0±5.2
ALT (U/L)	38.0(23.0–66.0)
AST (U/L)	56.0(60.0–87.0)
Total bilirubin (*μ*mol/L)	96.0(61.0–203.0)
INR	1.60±0.39
Serum Cr (mmol/L)	72.0(60.0–99.0)
HBsAg, positive	75
HBeAg, positive	44
HBcAb IgM, positive	0
Cystatin C (mg/L)	1.06(0.90–1.48)
MELD score	17.4(13.7–21.4)
Child-Pugh score	10.0(9.0–11.0)
Modes of decompensation	10.0(9.0–11.0)
HE, n (%)	2(2.7%)
HRS, n (%)	5(6.7%)
Ascites, n (%)	48(64.0%)
Variceal bleeding, n (%)	22 (29.3%)

Data are expressed as *n*, mean ± SD, or median (interquartile range).

ALT, alanine aminotransferase; AST, aspartate aminotransferase; INR, international normalized ratio; Cr, creatinine; HE, hepatic encephalopathy; HRS, hepatorenal syndrome; MELD score, model for end-stage liver disease score.

**Table 2:** Comparison of the surviving and nonsurviving patients with HBV-DeCi.

	Non-surviving patients	Surviving patients	*P*
	(n = 25)	(n = 50)	
Age (years)	53.8±12.3	52.3±10.2	0.262
Gender (male/female)	19/6	40/10	0.768
Total protein (g/L)	58.5±9.3	58.9±7.5	0.199
Albumin (g/L)	28.1±5.1	27.9±5.3	0.873
ALT (U/L)	39.0(23.8-49.8)	37.0(21.0-74.0)	0.942
AST (U/L)	56.5(38.5-95.5)	53.0(40.8-86.5)	0.660
Total bilirubin (*μ*mol/L)	96.0(74.8-247.3)	101.0(51.0-180.0)	0.261
Serum Cr (mmol/L)	97.0(69.0-125.5)	66.5(58.0-83.0)	0.002
INR	1.79±0.49	1.59±0.30	0.040
MELD score	20.5(17.9-22.7)	15.2(13.0-17.8)	<0.001
Child-Pugh score	10.0(9.0-11.0)	10.0(9.0-10.0)	0.376
Cystatin C (mg/L)	1.52(1.12-2.12)	0.98(0.87-1.18)	<0.001

Data are expressed as n, mean ± SD, or median (interquartile range).

ALT, alanine aminotransferase; AST, aspartate aminotransferase; Cr, creatinine; INR, international normalized ratio; MELD score, model for end-stage liver disease score.

**Table 3:** Multivariate analysis to identify the independent factors associated with outcomes in all patients with HBV-DeCi.

	Univariable			Multivariable		
	Odds ratio	95% CI	P	Odds ratio	95% CI	P
Cystatin C (mg/L)	26.293	5.189-133.216	<0.001	15.053	2.779-81.566	0.002
MELD score	1.271	1.112-1.454	<0.001	1.193	1.021-1.394	0.026
Age (year)	0.995	0.951-1.040	0.814			
Albumin (g/L)	0.980	0.893-1.075	0.664			
Child-Pugh score	1.434	0.949-2.166	0.087			

MELD score, model for end-stage liver disease score.

**Table 4:** Multivariate analysis to identify the independent factors associated with outcomes in patients with HBV-DeCi who had normal serum Cr levels.

	Univariable			Multivariable		
	Odds ratio	95% CI	P	Odds ratio	95% CI	P
Cystatin C (mg/L)	18.993	2.515-143.461	0.001	12.860	1.560-106.051	0.004
Age (year)	0.991	0.934-1.052	0.775			
Albumin (g/L)	0.999	0.876-1.141	0.994			
Child-Pugh score	1.427	0.830-2.454	0.199			
MELD score	1.244	1.042-1.484	0.007			

MELD score, model for end-stage liver disease score.

## Data Availability

The data used to support the findings of this study have not been made available in order to protect patient privacy.

## Abbreviations

ALT: Alanine aminotransferase
AST: Aspartate aminotransferase
AUCs: Areas under the curve
Cr: Creatinine
DeCi: Decompensated cirrhosis
GFR: Glomerular filtration rate
HBV: Hepatitis B virus
HE: Hepatic encephalopathy
HRS: Hepatorenal syndrome
INR: International normalized ratio
MELD score: Model for end-stage liver disease score
ROC: Receiver operating characteristic.
